# Potential Markers of Neurocognitive Disorders After Cardiac Surgery: A Bibliometric and Visual Analysis

**DOI:** 10.3389/fnagi.2022.868158

**Published:** 2022-06-01

**Authors:** Linna Ji, Fang Li

**Affiliations:** Department of Neurology, Fu Xing Hospital, Capital Medical University, Beijing, China

**Keywords:** markers, perioperative neurocognitive disorders, postoperative cognitive dysfunction, postoperative delirium, CiteSpace, scientometrics

## Abstract

**Background:**

Identifying useful markers is essential for diagnosis and prevention of perioperative neurocognitive disorders (PNDs). Here, we attempt to understand the research basis and status, potential hotspots and trends of predictive markers associated with PNDs after cardiac surgery via bibliometric analysis.

**Methods:**

A total of 4,609 original research articles and reviews that cited 290 articles between 2001 and 2021 were obtained from the Web of Science Core Collection (WoSCC) as the data source. We used the software CiteSpace to generate and analyze visual networks of bibliographic information, including published years and journals, collaborating institutions, co-cited references, and co-occurring keywords.

**Results:**

The number of annual and cumulative publications from 2001 to 2021 has been increasing on the whole. The Harvard Medical School was a very prolific and important institution in this field. The journal of Ann Thorac Surg (IF 4.33) had the most publications, while New Engl J Med was the most cited journal. Neuron-specific enolase (NSE), S100b and kynurenic acid (KYNA) were frequently discussed as possible markers of PNDs in many references. Cardiopulmonary bypass (CPB) was a keyword with high frequency (430) and sigma (6.26), and inflammation was the most recent burst keyword.

**Conclusion:**

Potential markers of PNDs has received growing attention across various disciplines for many years. The research basis mainly focuses on three classic biomarkers of S100b, NSE, and KYNA. The most active frontiers are the inflammation-related biomarkers (e.g., inflammatory cells, cytokines, or mediators) and surgery-related monitoring parameters (e.g., perfusion, oxygen saturation, and the depth of anesthesia).

## Introduction

Neurocognitive disorders (NCDs), one of the most common complications after cardiac surgery, has long been a question of great interest in a wide range of fields (Gao et al., 2005; Lingehall et al., 2017; O'Brien et al., 2017). With the development in surgical procedures and anesthesia techniques, cardiac surgery is becoming much safer than ever before and fewer patients die from normal operation. Unfortunately, despite these improvements, neurocognitive complications such as delirium or cognitive decline still occasionally happen, particularly in the elderly with numerous comorbidities (Jensen et al., 2008; Kotfis et al., 2018). The reported incidences of them vary dramatically depending on different study populations, evaluation time, assessment tools and forth (Selnes et al., 2009; Langer et al., 2019; Subramaniam et al., 2019). Previous researches have shown that these complications tend to be associated with longer hospital stays, higher readmission rates, added economic costs, decreased living quality, increased mortality, and a possible increased risk for developing Alzheimer's disease (AD) (Kirfel et al., 2021; Pereira et al., 2021). Therefore, it should be of paramount importance to explore exact mechanisms and effective therapies of perioperative neurocognitive disorders (PNDs), an overarching term recommended by Evered et al. (2018).

In order to improve identification and prevention of these neurocognitive sequelae, many researchers have been seeking potential markers with high sensitivity and specificity (Alifier et al., 2020; Szwed et al., 2020; Du et al., 2021). As an objective parameter, markers can capture complex pathophysiological changes of bodies, assist in risk stratification and prognosis monitoring, and facilitate diagnosis and assessment of PNDs. To date, extensive studies have investigated related makers of patients who suffered PNDs after cardiac surgery, involving stress, trauma, inflammation, metabolism, endocrine and so on (Luo et al., 2021). However, the results are still controversial, and there is no general agreement about which useful marker should be chosen in clinical practice. To this end, it is necessary to sort out critical research findings on makers of PNDs after cardiac surgery for the purpose of better further studies.

CiteSpace, a web-based Java application for bibliometric analysis and visualization, was developed in 2004 by Professor Chen (2004). It takes a set of bibliographic records as its input and generate a synthesized network based on multiple individual networks derived from each year's literature. CiteSpace allows users to analyze the intellectual structure of different knowledge networks visually, including collaboration network, co-occurrence network, and co-citation networks of cited authors, journals, or references (Chen and Song, 2019). Compared with traditional systematic reviews, this intuitive and flexible approach can provides a valuable overview of how a scientific field has been evolving over time and helps researchers to track the development of some areas they are really interested in Chen (2017).

Here, this review set out to detect the research basis, hotspots and frontiers of potential markers associated with PNDs after cardiac surgery through quantitative analysis of vast academic documents.

## Materials and Methods

### Search Strategy

Relevant publications between 2001 and 2021 were collected from the Science Citation Index Expanded (SCI-E) of the Web of Science Core Collection (WoSCC). The search strategy was “TS = (heart or cardiac or cardio^*^) AND TS = (surgery) AND TS = (cognition or ^*^cognitive or delirium) AND TS = (^*^marker^*^).” The initial topic search resulted in 290 records. We expanded the dataset by citation indexing and found 5,109 citing original research articles and review articles in English between 2001 and 2021. After preprocessing by CiteSpace to remove duplicates and rejects, 4,609 records were identified and used in the subsequent analysis. The detailed search strategy is presented in Supplementary Figure 1. All data were retrieved and downloaded in August 21, 2021.

### Data Analysis

A new version of CiteSpace (v5.8.R3) and Excel (2019) was used to visualize and analyze the dataset. The knowledge maps formed by CiteSpace consist of nodes and links. The size of nodes in a map represents frequency of different items such as author, institution, and country, and links between nodes represent the strength of the cooperation, co-occurrence, and co-citation relationships. Several commonly used structural metrics of CiteSpace are betweenness centrality, burstness and sigma score. Nodes with high betweenness centrality are likely to sit in the middle of two large groups of nodes which are usually regarded as turn points in a field. The intensity and duration of a citation burst indicate the degree of abrupt changes in the scientific community caused by a specific publication or keyword. If a node is strong in both centrality and burstness, it will have a high sigma score. In a map, a purple ring and red ring of a node mean that it has high betweenness centrality and strong citation burst (Chen et al., 2012).

## Results and Discussion

### Year, Institutions of Publication

**Figure 1:**
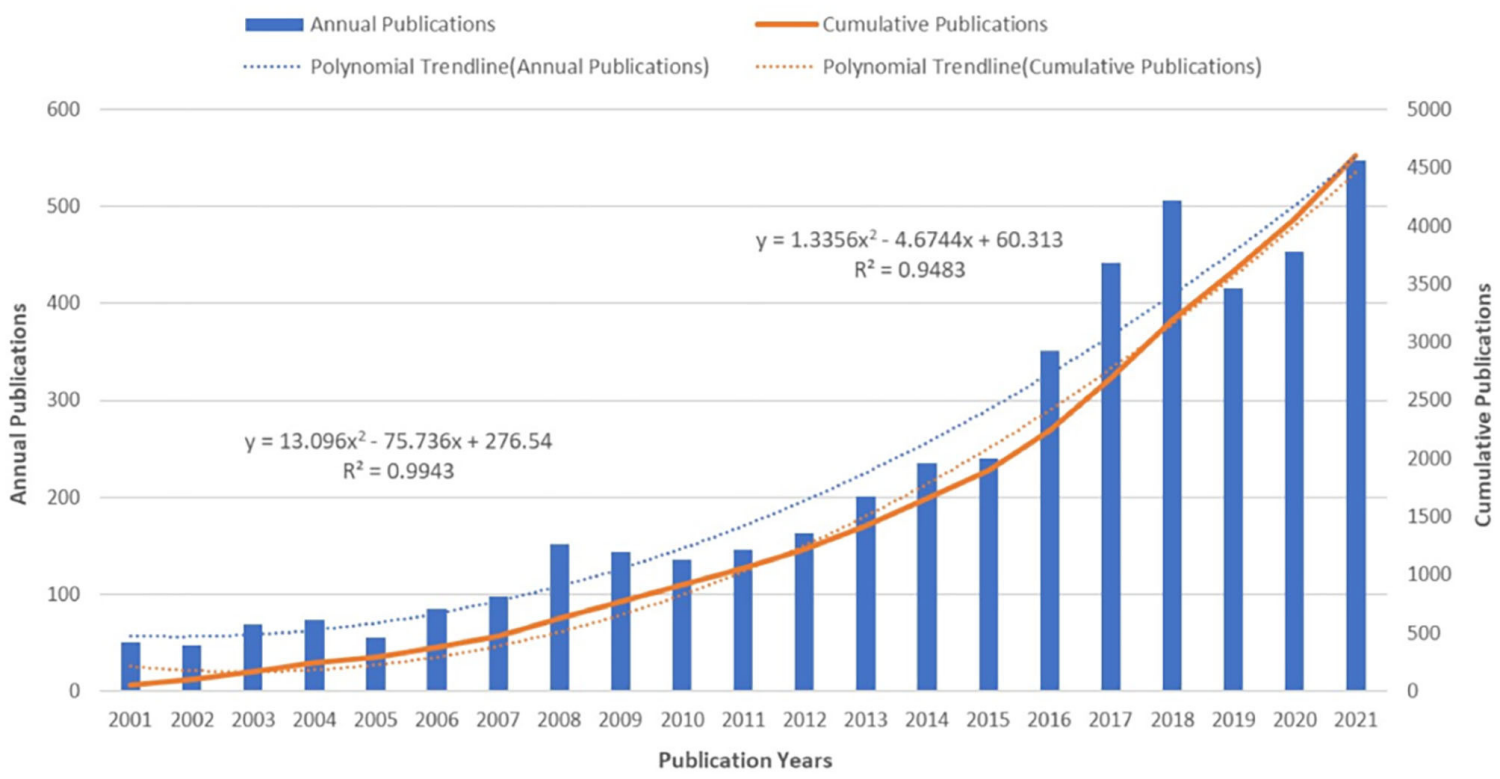
The number of annual publications and growth trends on makers of PNDs after cardiac surgery from 2001 to 2021.

In Figure 1, there was a clearly upward trend of the cumulative publications between 2001 and 2021, which increased from 50 to 4,609. The polynomial trendlines fitted the annual and cumulative number of them well (*R*^2^ = 0.9483, 0.9943). Although the annual number in 2019 and 2020 were slightly less than previous 2 years, the number of publications in 2021 was 547 up to now, which was the most productive year over the past two decades. These results indicate that the issue of markers associated with PNDs after cardiac surgery has received continuous attention and extensive research is being conducted recently.

As shown in Table 1, the top five prolific institutions were Duke University (102), Harvard Medical School (101), University of Pennsylvania (100), University of Toronto (94), Johns Hopkins University (89). As regards sigma score, the lead institution was Harvard University (3.09), followed by the Harvard Medical School (1.56), King's College London (1.37), University College London (1.31), and Yale University (1.28). Most of them are American institutions, suggesting that the United States is staying ahead in this research field, and is playing a vital role in the world. Though the number of articles published by many academic institutions from England or other countries was fewer than those in US, these institutions are active in multi-institutional cooperation and have still produced many groundbreaking research outcomes.

**Table 1:** Top 15 institutions distributed by publications and sigma.

**Rank**	**Institution**	**Publications**	**Original country**	**Institution**	**Sigma**	**Original country**
1	Duke Univ	102	United States	Harvard Univ	3.09	United States
2	Harvard Med Sch	101	United States	Harvard Med Sch	1.56	United States
3	Univ Penn	100	United States	Kings Coll London	1.37	England
4	Univ Toronto	94	Canada	UCL	1.31	England
5	Johns Hopkins Univ	89	United States	Yale Univ	1.28	United States
6	Columbia Univ	81	United States	Univ Penn	1.21	United States
7	Harvard Univ	79	United States	Univ London Imperial Coll Sci Technol and Med	1.17	England
8	Univ Calif San Francisco	69	United States	Univ Bristol	1.16	England
9	Vanderbilt Univ	61	United States	Univ Amsterdam	1.15	Netherlands
10	UCL	60	England	Univ Pittsburgh	1.13	United States
11	Emory Univ	57	United States	Univ Cambridge	1.11	England
12	Capital Med Univ	52	China	Univ Copenhagen	1.11	Denmark
13	Univ Melbourne	51	Australia	Cincinnati Children's Hosp Med Ctr	1.1	United States
14	Univ Padua	49	Italy	Shanghai Jiao Tong Univ	1.1	China
15	Univ Pittsburgh	49	United States	Univ Oxford	1.1	England

### Journals and Co-cited Journals

Journals are the main carrier of scientific research results, through which we can know the widespread distribution and dissemination of the relevant literature in a given field. Looking at Table 2, the Ann Thorac Surg (IF 4.33) had the most publications, followed by J Cardiothor Vasc An (IF 2.628), Anesth Analg (IF 5.178), J Thorac Cardiov Sur (IF 5.209), and Brit J Anaesth (IF 9.166). The Ann Thorac Surg is a professional peer-reviewed medical journal intended for promoting scholarship in cardiothoracic surgery patient care, clinical practice, research, education, and policy. According to the Journal Citation Report (JCR) on the Web of Science (WOS), the journal has 509 publications, with 41,620 citations in 2020. It ranked 36 out of 211 in surgery and 51 out of 142 in cardiovascular system.

**Table 2:** Top 15 journals that published articles on makers of PNDs after cardiac surgery.

**Rank**	**Publications**	**Journal**	**IF (2020)**
1	117	Ann Thorac Surg	4.330
2	100	J Cardiothor Vasc An	2.628
3	88	Anesth Analg	5.178
4	88	J Thorac Cardiov Sur	5.209
5	80	Brit J Anaesth	9.166
6	67	Eur J Cardio-Thorac	4.191
7	58	PLoS ONE	3.240
8	57	Anesthesiology	7.892
9	42	Perfusion-UK	1.972
10	39	BMC Anesthesiol	2.217
11	39	Heart Surg Forum	0.676
12	33	Front Aging Neurosci	5.750
13	33	Sci Rep-UK	4.380
14	32	Acta Anaesth Scand	2.105
15	32	Curr Opin Anesthesio	2.706

As can be seen from the Table 3, the top five co-cited journals were New Engl J Med, Lancet, Circulation, Ann Thorac Surg, Anesthesiology and the top five in terms of sigma were Am J Clin Nutr, J Thorac Cardiov Sur, J Trace Elem Med Bio, Brain Res Bull, Obesity Facts. The fundamentals of the research field mostly came from above journals. Among the top 15 co-cited journals, New Engl J Med, Lancet, JAMA-J Am Med Assoc are top journals in medical area, and the rest are the most important journals in the fields of anesthesiology and cardiothoracic surgery. In addition, from the top journals ranked by sigma score we can know that part of articles also involved the nutrition, endocrinology, molecular and cellular mechanisms associated with cognition and other brain functions.

**Table 3:** Top 15 co-cited journals distributed by citations and sigma.

**Rank**	**Journal**	**Citations**	**Journal**	**Sigma**
1	New Engl J Med	2,116	Am J Clin Nutr	3.09
2	Lancet	1,849	J Thorac Cardiov Sur	2.5
3	Circulation	1,678	J Trace Elem Med Bio	2.43
4	Ann Thorac Surg	1,639	Brain Res Bull	1.77
5	Anesthesiology	1,520	Obesity Facts	1.56
6	JAMA-J Am Med Assoc	1,487	Ann Thorac Surg	1.56
7	J Thorac Cardiov Sur	1,442	Brain Res	1.52
8	PLoS ONE	1,368	World J Surg	1.48
9	Anesth Analg	1,250	Med Educ	1.45
10	Brit J Anaesth	1,221	Neurosurgery	1.44
11	Stroke	1,139	Chest	1.42
12	Eur J Cardio-Thorac	1,030	New Engl J Med	1.41
13	Crit Care Med	988	Am J Surg	1.34
14	J Am Coll Cardiol	978	J Trauma	1.34
15	J Cardiothor Vasc An	913	Cardiovasc Surg	1.32

### Co-cited References Analysis

Co-cited references are the theoretical basis of a research field, the most cited of which can be seen as hotspots and landmarks because they have attracted much attention and made great contributions to the field. The most cited articles in each year between 2001 and 2021 were chosen in accordance with g-index (Egghe, 2006) to construct a year's individual network.

#### Landscape View

**Figure 2:**
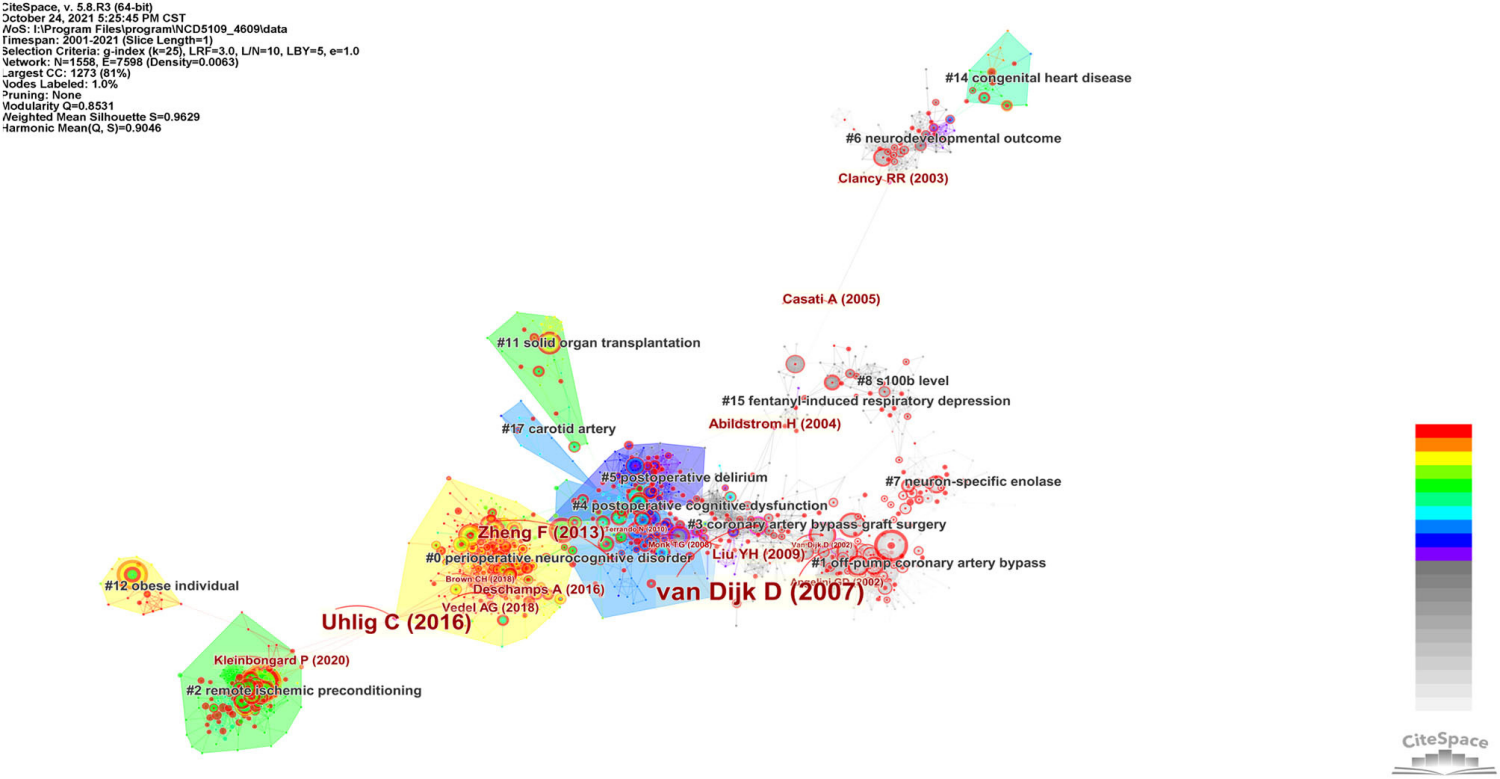
A landscape view of the reference co-citation network.

The integrated reference co-citation network was shown in Figure 2. It contained 1,558 nodes, 7,598 linkes, and 150 clusters. The modularity was 0.8531, which means that the structure of the network is clear and well-organized. The silhouette score of 0.9629 was very high, suggesting the clustering result was highly reliable. Each tree ring of a node represents the citation history of an article. Its size, color and thickness imply the cited times, year of publication and citations in a single time slice, respectively. What stand out in this figure were the references with high centrality, such as “van Dijk et al. (2007),” “Uhlig et al. (2016),” “Zheng et al. (2013),” “Liu et al. (2009),” and “Abildstrom et al. (2004)”. They were key hubs connecting different groups of nodes, which can be regarded as structurally essential references in this network. The color bar, ranging from cold colors to warm colors, indicated a specified period starting from 2001 to 2021. For example, lines or areas in purple generated in 2012, were earlier than those in red or orange.

**Figure 3:**
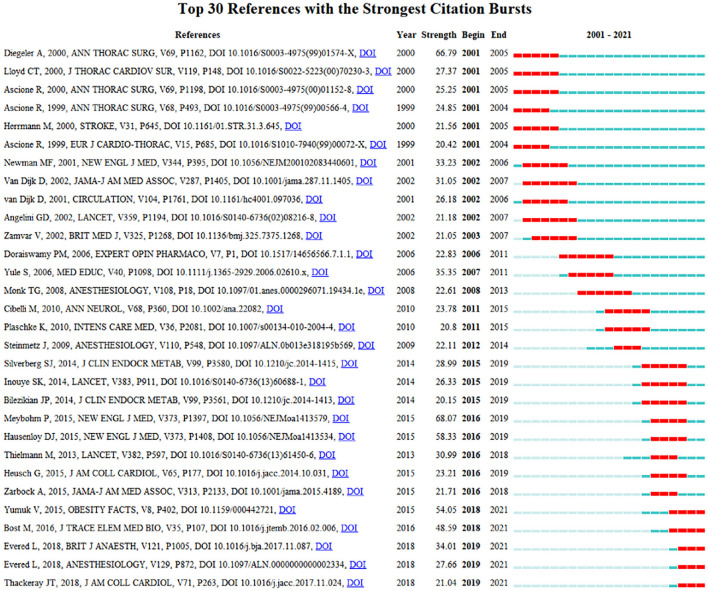
Top 30 references with strong citation bursts.

The top 30 references with strong citation bursts can be grouped by the beginning time of burst (Figure 3). Instead of introduced all the 30 references, the reference with the strongest citation burst of PNDs in each group will be discussed as the most representative article. Among citation bursts starting in 2001 and 2002, the strongest bursts are associated with a 2000 paper by Diegeler et al. and a 2001 paper by Newman et al. Coronary artery bypass grafting (CABG) is a well-established surgical procedure for heart disease and can be performed with or without cardiopulmonary bypass (CPB), namely on- or off-pump CABG. Diegeler et al. (2000) investigated the impact of CPB on neuropsychological function of patients who underwent on-pump and off-pump CABG using transcranial Doppler ultrasound (TCD) and S-100 analytical method. Around the same year, some researchers focused on the role of neurobiochemical markers of brain damage and the change of inflammatory response during cardiac surgery. In 2001, Newman et al. (2001) undertook a prospective study to confirm the significant effect of perioperative cognitive decline after CABG on long-term cognitive function 5 years later. Since then, many randomized controlled trials (RCTs) have compared cognitive outcomes in different postoperative stages after on- vs. off-pump CABG.

In order to avoid possible confounding of those adverse events in surgeries caused by failed non-technical operations, the article published by Yule et al. (2006) developed a behavior rating system specifically for surgeons' non-technical skills (e.g., awareness, communication, and teamwork), aiming to allow surgeons to structure observations and improve safety and efficiency of surgical performances further.

Cibelli, Fidalgo, Terrando, Ma, Monaco and Feldmann (2010) published in 2010 suggested that inflammation plays a prominent role in the pathogenesis of postoperative cognitive dysfunction (POCD) using a mouse model of orthopedic surgery. Citation bursts starting in 2016 and 2018 are, respectively, led by Meybohm et al. (2015) and Yumuk et al. (2015) article published in 2015. The former was a prospective, double-blind, multicenter, randomized, controlled trial aiming to prove whether remote ischemic preconditioning (RIPC) can improve clinical outcomes in patients undergoing elective cardiac surgery, while the latter provided many explicit guidelines for obesity management in adults. In 2018, Evered et al. (2018) revised the nomenclature for PNDs to enhance consistency of communication and reporting in this field. It has the strongest citation burst starting in 2019, and it is still going strong.

These articles, identified based on citation bursts, indicated that researchers primarily focused on the following issues in past 20 years: the assessment, comparison and definition of neurocognitive outcomes in all stages during cardiac surgery, the predictive value of markers and tools in PNDs such as trauma-related or inflammation-related substances, the prevention and treatment of PNDs such as performing RIPC or managing high-risk population groups. In the different period, there are different focuses on this topic.

#### Timeline View

**Figure 4:**
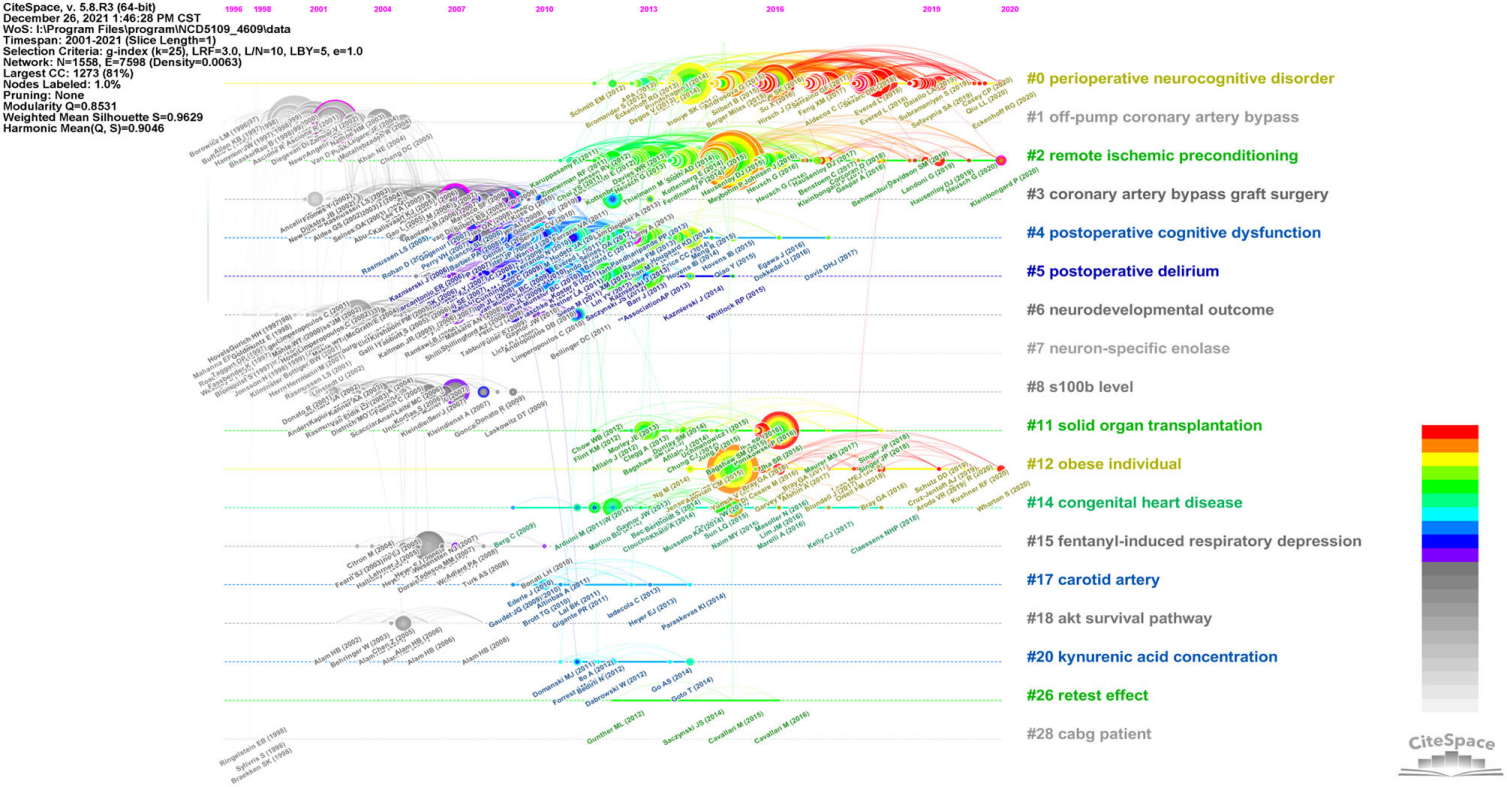
A timeline visualization of the largest clusters.

Each cluster is displayed as a horizontal timeline from left to right. The three most cited references in every year are listed below each timeline. Figure 4 lists the first 18 large clusters and demonstrates that Cluster #0 is the largest cluster and it is active in recent years. The field's focus has been gradually expanded from Cluster #5 postoperative delirium (POD) and Cluster #4 POCD to the Cluster #0 PNDs. Interestingly, remote ischemic preconditioning and obese individual are another two active clusters now. This is probably a hint that we can try to investigate the level of specific markers in different conditions or population.

In these timelines, there were three clusters labeled with specific markers, which is Cluster #7, #8, and #20. Although they were neither the largest clusters nor the most active fields, they were closely associated with our purpose to explore the potential markers of PNDs. It was much important for us to understand how these markers work and why they were studied in this field.

S100b and Neuron-specific enolase (NSE) were both first described in 1965 (Moore and McGregor, 1965), while kynurenic acid was initially found as a constituent of canine urine in 1853 (Liebig, 1853). In 2001, Donato (2001) published a paper in which he demonstrated that S100b is a member of a multigenic family of Ca2+-modulated proteins of the EF-hand type. It not only acts as an intracellular regulator to modulate cell proliferation, migration and differentiation, but can be seen as an extracellular signal playing a neurotrophic or neurotoxic role under different conditions (Donato et al., 2009). Additionally, Kleindienst et al. (2007) analyzed the value of S100b as a marker of brain damage and possible treatment option, and they concluded that the positive effect of S100b on cognition and neurogenesis can improved the functional prognosis of patients with brain injury, which probably had a therapeutic potential. But it should be noted that serum S100b levels can't reflect the corresponding cerebral S100b release.

NSE, a glycolytic enzyme found in the neuronal cytoplasm and involved in the energy metabolic process, has been widely studied in many nervous system diseases. Neuronal damage or death can increases its levels in cerebrospinal fluid (CSF) or serum (Marangos and Schmechel, 1987; Rasmussen et al., 1999). Herrmann et al. (2000) reported that postoperative serum concentrations and kinetics of S100b and NSE after cardiac surgery not only reflected early different neuropsychological outcomes but also might mirror underlying damage to neuronal and glial brain tissue. As a metabolite of tryptophan produced in the kynurenine pathway, kynurenic acid (KYNA) possesses neuroactive function, which may influence important neurophysiological and neuropathological processes. Forrest et al. (2011) reported that KYNA was a significant correlate of performance in the Stroop C and Trail A and B tests, and predicted cognitive function in patients following cardiac surgery. However, due to their poor specificity, inadequate sensitivity and inaccurate assay methods (Smith et al., 2019), the correlations between these biomarkers and postoperative cognitive function have been controversial.

#### Cluster Analysis

Nine major clusters were listed in the Table 4. The titles of citing articles were extracted to label the corresponding clusters, which are generally considered as the research fronts of these clusters. All labels chosen by the log-likelihood ratio test method (LLR) were used for analysis. Of those nine clusters, Cluster #7 and #8 have been mentioned in Section Timeline View, while other clusters such as Cluster #1, #2, #3, and #6 were related to operation ways and neurodevelopmental problems. Hence, We selectively interpreted the Cluster #0 PNDs, #4 POCD, #5 POD in detail since they were directly related to neurocognitive disorders after cardiac surgery, the topic we're interested in. Besides, in each cluster, we emphatically discussed those references with high citations and high topic relevance.

**Table 4:** Major clusters of co-cited references.

**Cluster ID**	**Size**	**Silhouette**	**Mean (year)**	**Label (LLR)**
0	257	0.971	2016	Perioperative neurocognitive disorder
1	195	0.961	2000	Off-pump coronary artery bypass
2	148	0.99	2014	Remote ischemic preconditioning
3	136	0.916	2006	Coronary artery bypass graft surgery
4	114	0.923	2011	Postoperative cognitive dysfunction
5	107	0.957	2010	Postoperative delirium
6	78	0.993	2004	Neurodevelopmental outcome
7	58	0.982	1998	Neuron-specific enolase
8	57	0.934	2004	s100b level

As Table 5 shows, the top 10 cited references in Cluster #0 were mostly published in the past 5 years. Inouye et al. (2014), the most cited reference in this cluster, summarized the epidemiology, etiology, diagnosis, evaluation and management of delirium in elderly people. It offered many potential pathophysiologic contributors to delirium, including neurotransmitters (e.g., acetylcholine, melatonin, and serotonin), inflammation (e.g., IFN α/β, IL-6/8/10, and TNF-α), physiological stressors (e.g., cortisol and neopterin), electrolyte disorders (e.g., sodium, calcium, and magnesium), metabolic derangements (e.g., lactate, glucose, and IGF-1) and so on. More specifically, Hughes et al. (2020), the citing article with coverage of 34%, provided evidence-based consensus statements and practice recommendations regarding POD identification and prevention, based on an established iterative Delphi process and Grading of Recommendations Assessment, Development and Evaluation (GRADE) Criteria in the Sixth Perioperative Quality Initiative (POQI-6) consensus conference.

**Table 5:** Top 10 cited references and citing articles of Cluster #0 perioperative neurocognitive disorder.

**Cited references**		**Citing articles**	
**Cites**	**Author (year) journal, volume, page**	**Coverage %**	**Author (year) title**
90	Inouye et al. (2014) Lancet, V383, P911	34	Hughes et al. (2020) American society for enhanced recovery and perioperative quality initiative joint consensus statement on postoperative delirium prevention
70	Evered et al. (2018) Brit J Anaesth, V121, P1005	26	Safavynia and Goldstein (2019) The role of neuroinflammation in postoperative cognitive dysfunction: moving from hypothesis to treatment
57	Evered et al. (2018) Anesthesiology, V129, P872	24	Berger et al. (2018) Neurocognitive function after cardiac surgery from phenotypes to mechanisms
50	Hirsch et al. (2016) J Neuroinflamm, V13, P0	23	Jin et al. (2020) Postoperative delirium: perioperative assessment, risk reduction, and management
45	Aldecoa et al. (2017) V34, P192	20	Subramaniyan and Terrando (2019) Neuroinflammation and perioperative neurocognitive disorders
44	Su et al. (2016) Lancet, V388, P1893	19	White et al. (2019) Guidelines for the peri-operative care of people with dementia: guidelines from the association of anesthetists
43	Inouye et al. (2016) Alzheimers Dement, V12, P766	18	Sanjanwala et al. (2020) Delirium prevention in postcardiac surgical critical care
42	Hovens et al. (2016) Brain Behav Immun, V54, P178	18	Wilson et al. (2020) delirium
41	Evered et al. (2016) Anesthesiology, V124, P353	14	Yang et al. (2020) neuroinflammation after surgery: from mechanisms to therapeutic targets

Evered et al. (2018), the second most cited reference, recommended using PNDs to describe cognitive impairment identified in the perioperative period, which includes pre-existing mild or major neurocognitive disorders (NCD) before operation, POD after surgery, delayed neurocognitive recovery within postoperative 30 days, and mild or major POCD from postoperative 30 days to 12 months. Both of cited references are major milestones in Cluster #0. Other citing articles mainly focused on the assessment, prevention and comprehensive management of PNDs, especially the part of neuroinflammation-related medicators and pathways.

In CiteSpace, key concepts can be algorithmically extracted from the titles of citing articles in Cluster #0 (Supplementary Figure 2). When it comes to potential diagnostic or prognostic indicators, some of them referred to certain soluble molecular substances in biological fluids such as serum, plasma or CSF, mostly associated with inflammation, brain injury, stress response, metabolism, neurotransmitter or key regulators in these processes, such as C-reactive protein (CRP), glial fibrillary acidic protein (GFAP), visinin-like/galectin-3 protein.

In Figure 4, some of more recent and highly cited members in Cluster #0 include a review of different experimental approaches and models that have been used in preclinical postoperative cognitive dysfunction research (Eckenhoff et al., 2020), a prospective biomarker cohort study of POD is associated with increased plasma neurofilament light (NfL) (Casey et al., 2020), and a mechanism research of BDNF/TrkB signaling disruption mediated by NMDAR/Ca2+/calpain might contribute to POCD in aging mice (Qiu et al., 2020).

Cited references and citing articles of Cluster #4 POCD were listed in Table 6. van Harten et al. (2012) suggested that more biochemical markers specific to neuronal damage should be identified, such as glial fibrillary-associated protein and brain- and heart-type fatty acid binding protein. The five citing articles are all reviews, which largely summarized many common studied markers associated with Alzheimer's disease pathophysiology (e.g., ApoE, Aβ1-42, and p-tau), inflammatory process (e.g., NF-kappaB, MMP9, and MCP-1), neuronal injury. Androsova et al. (2015) emphasized the importance of systems biology methods encompassing experimental measurements, imaging techniques and mathematical/computational modeling, so that we can not only understand the pathomechanisms of PNDs at different levels, but also integrate sparse markers into a complete knowledge network through some known molecular interactions and pathways. In this way, more potential markers and underlying pathways can be found easier than ever before with purposeful design.

**Table 6:** Top 5 cited references and citing articles of Cluster #4 postoperative cognitive dysfunction.

**Cited references**		**Citing articles**	
**Cites**	**Author (year) journal, volume, page**	**Coverage %**	**Author (year) title**
48	Cibelli et al.'s (2010) Ann Neurol, V68, P360	38	Nadelson et al. (2014) Perioperative cognitive trajectory in adults
45	Monk et al. (2008) Anesthesiology, V108, P18	25	Berger et al. (2014) Alzheimer's disease, anesthesia, and surgery: aclinically focused review
43	Steinmetz et al. (2009) Anesthesiology, V110, P548	21	Androsova et al. (2015) Biomarkers of postoperative delirium and cognitive dysfunction
40	Chan et al. (2013) J Neurosurg Anesth, V25, P33	19	Hussain et al. (2014) General anesthetic and the risk of dementia in elderly patients: current insights
40	van Harten et al. (2012) Anesthesia, V67, P280	19	McDonagh et al. (2014) Neurological complications of cardiac surgery

The most cited members of Cluster #5 represent important mileposts in relation to POD (Table 7), such as Saczynski et al. (2012) article on the cognitive trajectory after POD during the first year, and Lin, Chen and Wang (2012) article on the risk factors of delirium after cardiac surgery. The citing article with the highest citation coverage of 21% in this cluster, Maldonado (2013), summarized seven complementary theories and their interrelation: the hypotheses of “neuroinflammatory,” “neuronal aging,” “oxidative stress,” “neuroendocrine,” “diurnal dysregulation,” and “network disconnectivity” can acted together to lead to a final pathway associated with neurotransmitter alterations, which generally were deficiencies in acetylcholine (Ach) and melatonin; excess of dopamine (DA), norepinephrine (NE), and/or glutamate (GLU); and decrease or increase in serotonin (5HT), histamine, and/or γ-aminobutyric acid (GABA).

**Table 7:** Top 5 cited references and citing articles of Cluster #5 postoperative delirium.

**Cited references**		**Citing articles**	
**Cites**	**Author (year) journal, volume, page**	**Coverage %**	**Author (year) title**
52	Saczynski et al. (2012) New Engl J Med, V367, P30	21	Maldonado (2013) Neuropathogenesis of delirium: review of current etiologic theories and common pathways
44	Lin et al.'s (2012) J Cardiac Surg, V27, P481	20	Cunningham and Maclullich (2013) At the extreme end of the psychoneuroimmunological spectrum: Delirium as a maladaptive sickness behavior response
44	American Psychiatric Association (2013) Diagn Stat Man Ment, V0, P0	17	Guenther et al. (2013) Predisposing and precipitating factors of delirium after cardiac surgery: a pospective observational cohort study
42	Plaschke et al. (2010) Intens Care Med, V36, P2081	17	Girard et al. (2012) Associations of markers of inflammation and coagulation with delirium during critical illness
35	van Munster et al. (2010) Brain Cognition, V74, P18	17	de Rooij and van Munster (2013) Melatonin deficiency hypothesis in delirium: a synthesis of current evidence

#### Structural Variation Analysis

The structural variation analysis can identify the potentially transformative articles published in recent years according to their contributions to the global or local structure of the knowledge domain of interest. To great extent, the method compensates for the limitation of citation-based analysis relies on citations that are greatly influenced by the publication time of articles.

Table 8 lists the first five articles in each of the last 3 years, which were ranked by the harmonic mean (H) of three different structural variation variables, namely ΔM, ΔCLw, and C_KL_ (Chen, 2012). The five highly ranked articles in 2021 focused on the pathophysiologic mechanisms of PNDs, such as anesthetic regimen, cerebral perfusion pressure management, cerebral oximetry monitoring and so on (Akhtar et al., 2021; Evered and Goldstein, 2021; Pisano et al., 2021; Snyder et al., 2021; Zugni et al., 2021). In 2019 and 2020, there were two valuable articles providing evidence-based recommendations on perioperative delirium care and prevention (White et al., 2019; Hughes et al., 2020). Also, several articles studied related neurovascular and immune mechanisms of PNDs and their possible markers (Luo et al., 2019; Yang and Terrando, 2019; Fong et al., 2020; Majewski et al., 2020; Wang et al., 2020).

**Table 8:** Potentially transformative articles published in recent 3 years (2019–2021).

**Year**	**ΔM**	**ΔCL_**w**_**	**C_**KL**_**	**H**	**GC**	**Title**	**References**
2021	93.7353	−1.6773	0.2784	0.9979	1	The impact of anesthetic regimen on outcomes in adult cardiac surgery: a narrative review	Pisano et al., 2021
2021	93.7353	−2.2141	0.1829	0.5968	2	Cerebral hypoxia: its role in age-related chronic and acute cognitive dysfunction	Snyder et al., 2021
2021	83.294	−8.4738	0.1862	0.5698	1	Multicenter international survey on cardiopulmonary bypass perfusion practices in adult cardiac surgery	Akhtar et al., 2021
2021	93.7353	−6.919	0.1015	0.3087	0	Noninvasive neuromonitoring in the operating room and its role in the prevention of delirium	Zugni et al., 2021
2021	93.3176	−5.4489	0.0644	0.1954	2	Reducing perioperative neurocognitive disorders (pnd) through depth of anesthesia monitoring: a critical review	Evered and Goldstein, 2021
2020	94.3856	−3.1238	0.0395	0.1200	0	Delirium after tavr: crosspassing the limit of resilience	van der Wulp et al. (2020)
2020	91.5785	−7.4362	0.029	0.0873	3	Association of plasma neurofilament light with postoperative delirium	Fong et al., 2020
2020	88.3034	−1.5263	0.028	0.0855	28	American society for enhanced recovery and perioperative quality initiative joint consensus statement on postoperative delirium prevention	Hughes et al., 2020
2020	94.1517	−2.5738	0.027	0.0818	13	Neurovascular and immune mechanisms that regulate postoperative delirium superimposed on dementia	Wang et al., 2020
2020	91.5785	−2.8884	0.0193	0.0583	1	Current evidence regarding biomarkers used to aid postoperative delirium diagnosis in the field of cardiac surgery-review	Majewski et al., 2020
2019	99.2729	0	0.5361	0	23	Guidelines for the peri-operative care of people with dementia guidelines from the association of anesthetists	White et al., 2019
2019	96.7425	0	0.2775	0	20	Postoperative cognitive dysfunction in the aged: the collision of neuroinflammaging with perioperative neuroinflammation	Luo et al., 2019
2019	96.7425	0	0.2513	0	8	Impact of postoperative dexmedetomidine infusion on incidence of delirium in elderly patients undergoing major elective noncardiac surgery: a randomized clinical trial	Sun et al. (2019)
2019	93.2134	0	0.1491	0	30	Lasting effects of general anesthetics on the brain in the young and elderly: “mixed picture” of neurotoxicity neuroprotection and cognitive impairment	Wu et al. (2019)
2019	93.2134	0	0.1441	0	1	The evolving role of specialized pro-resolving mediators in modulating neuroinflammation in perioperative neurocognitive disorders	Yang and Terrando, 2019

### Co-occurring Keywords Analysis

Keywords, usually concise and refined concepts, are the core topics of academic articles. In CiteSpace, co-occurring keywords reflect research hotspots and focus points in a field. The top 15 keywords were ranked by frequency and sigma in Table 9. In addition to search terms of “cardiac” and “surgery”, CPB was the keyword with highest frequency (430) and sigma (6.26). It is a technique commonly utilized in cardiac surgery to temporarily maintain the function of the heart and lungs, which was first performed by Dr. John H. Gibbon, Jr in May 1953 (Bauer and Tchantchaleishvili, 2018).

**Table 9:** Top 15 keywords ranked by frequency and sigma.

**Rank**	**Keywords**	**Frequency**	**Keywords**	**Sigma**
1	Cardiac surgery	619	Cardiopulmonary bypass	6.26
2	Surgery	466	Neuron specific enolase	2.25
3	Cardiopulmonary bypass	430	Coronary artery bypass	2.05
4	Outcome	357	Off pump	1.9
5	Risk factor	302	On pump	1.74
6	Dysfunction	275	Inflammatory response	1.64
7	Risk	267	Hypothermic circulatory arrest	1.56
8	Alzheimer's disease	256	Damage	1.5
9	Mortality	240	Stroke	1.41
10	Disease	236	Beating heart	1.4
11	Brain	213	Marker	1.37
12	Elderly patient	196	Performance	1.37
13	Association	189	Cerebral blood flow	1.33
14	Postoperative delirium	180	Release	1.32
15	Injury	177	Cerebrospinal fluid	1.31

Seeing from the Table 9, several major issues during cardiac surgery can be identified:

(1) Operation and Prognosis: According to a systematic review on clinical outcomes of CABG by Møller et al. (2008), opinions differed as to whether on-pump or off-pump surgery can offer patients better outcomes. They included 66 randomized trials and found no convincing evidence that off-pump surgery reduces any of the major clinical outcomes such as death, myocardial infarction, stroke, coronary reintervention except for atrial fibrillation. In 2017, Filardo et al. (2018) published a meta-analysis assessing efficacy [randomized controlled trials (RCTs)] and effectiveness (observational studies) of on- vs. off-pump CABG, and concluded that off-pump CABG has greater operative safety than the former but may harm lasting survival gains of patients.
(2) Neurologic Complications and Risk factors: Throughout the history of cardiac surgery and anesthesia, neurologic complications have been a primary concern, for instance, early or late stoke, delirium, cognitive decline and even dementia (Bedford, 1955; Gilman, 1965; Eagle et al., 1999; McDonagh et al., 2014). Thus, a bulk of literature have explored risk factors associated with these adverse events (Hedberg et al., 2011; Hood et al., 2018; Gaudino et al., 2019; Greaves et al., 2020), mainly divided into several categories: baseline patient characteristics (e.g., age, sex, genetics, physical fitness, multiple comorbidities, mental attitude, premorbid intelligence or cognitive function, alcohol, or drug abuse), surgery-related procedural variables (e.g., surgical approaches and duration, intraoperative monitoring and management of temperature, perfusion pressure, oxygen saturation, blood glucose and embolic load, anesthetic protocols, drugs, and dosage), and perioperative care (e.g., sedation, analgesia, mobilization, nutrition, and sleep enhancement). Also, it is of great significance for proposing effective prophylaxis to divide them into modifiable, partly modifiable and non-modifiable or pre, mid, and postoperative factors.
(3) Mechanisms and potential markers: The researches on these two aspects are essential and paramount for earlier diagnosis, prevention and treatment of PNDs. They can complement and reinforce each other, that is, the change of certain thing or index in an established mechanism may indicates its clinical application value as a predictive marker and vice versa. In a review, Berger et al. (2018) discussed six partly overlapped pathophysiologic mechanisms that may underlie PNDs: neuronal damage/aging, inflammation, embolism, cerebral autoregulation/oxygen delivery/temperature management, neurodegenerative disease pathology, and brain network dysfunction. Additionally, a growing body of literature has reported that gut microbiota might also play a critical role in PNDs (Xu et al., 2020). Therefore, a variety of markers can be found during these processes, which mainly consist of two types: biochemical markers and monitoring parameters, and two sources: biological fluids (CSF, blood, saliva, urine, sweat, and tears) and monitoring devices [magnetic resonance imaging (MRI), electroencephalographic (EEG), Near-infrared spectroscopy (NIRS), TCD, BIS monitor, Transesophageal echocardiography (TEE)].

**Figure 5:**
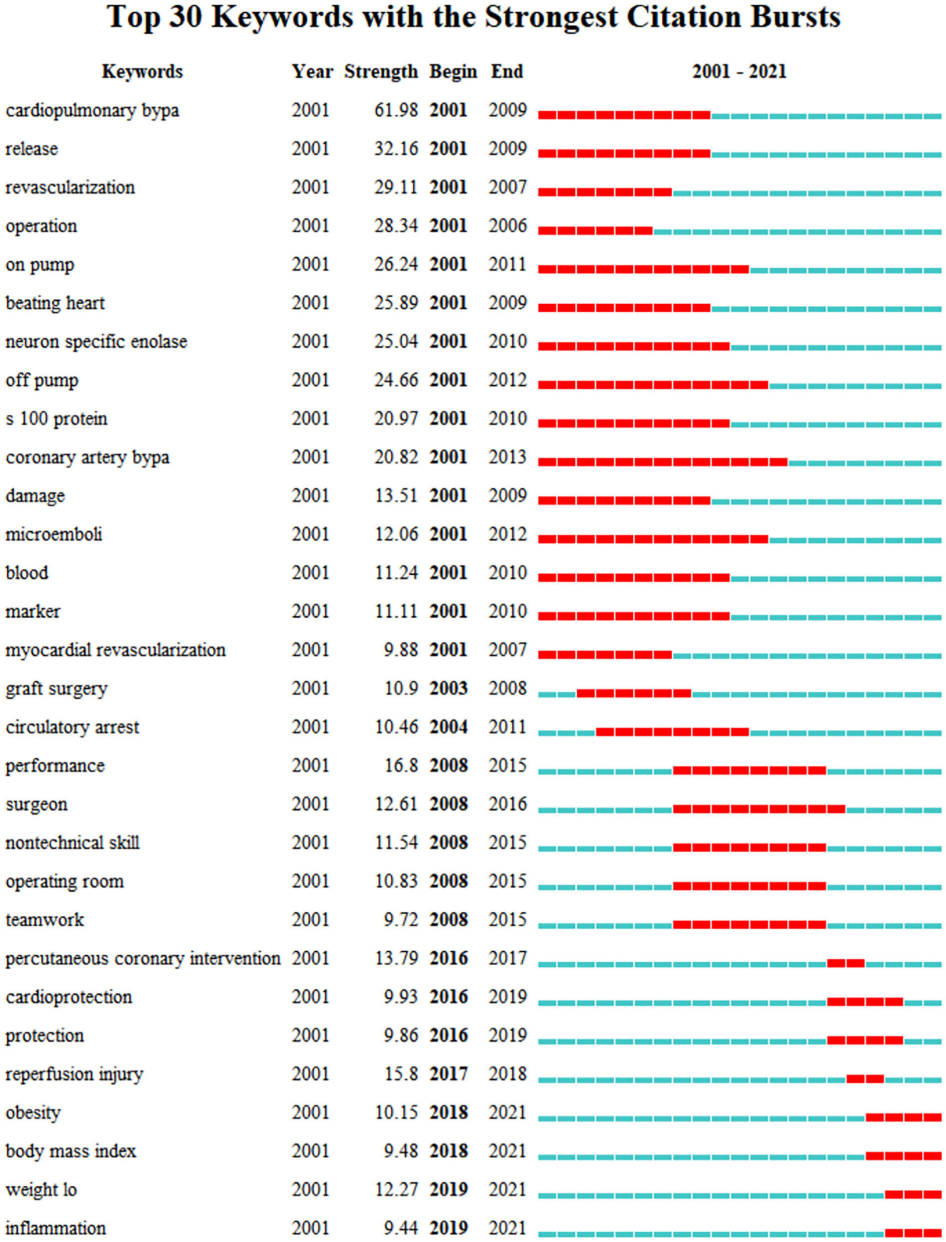
Top 30 keywords with strong citation bursts.

Figure 5 shows the top 30 keywords with the strongest bursts. The strongest ones include “cardiopulmonary bypass,” “release,” and “revascularization”. The most recent burst of keywords is “inflammation”, which is regarded as a research frontier in this field, and may well be studied intensively and deeply in the future. Briefly speaking, there are probably a couple of reasons for studying inflammation: on the one hand, inflammation can be regarded as a bridge connecting the cardiac surgery and neurocognitive disorders. Through upregulating cyclooxygenase 2 isozyme (COX-2) and matrix metalloproteinases (MMPs), peripheral pro-inflammatory cytokines can disrupt the integrity of the blood-brain barrier (BBB), allowing bone-marrow-derived monocytes (BMDM) to activate microglia cells and trigger neuroinflammation (Tan et al., 2021). Besides, Thackeray et al. (2018) reported that biphasic peaks of neuroinflammation in early acute and late chronic stages of cardiac injury were similar to a biphasic microglial activation pattern with peaks at initial onset of mild cognitive impairment and late advanced Alzheimer's disease. In addition, many animal studies have provided evidence that the anti-neuroinflammatory therapy after cardiac surgery can diminish microglial activation, attenuates neurogenesis suppression, and improve functional outcomes (Wang et al., 2022). And further research on clinical trials is still ongoing. On the other hand, with the advance of immunology, molecular biology, and examining techniques, researchers can consider questions from totally novel angles and merge a variety of accurate ways to explore potential inflammatory pathways and mechanisms in great depth.

### Limitations

Inevitably, there are several limitations in the study. Firstly, we subjectively chose specific search strategy and only analyzed the characteristics of those English publications retrieved from single database WOSCC. This probably let us ignore some valuable articles in other languages or data archives such as PubMed, Embase or MEDLINE. Secondly, we collected our data using general terms associated with this topic rather than POD or POCD and expanded the dataset through including lots of citing articles, which introduced many less relevant publications. Therefore, we selectively discussed significant articles and closely related clusters to make the interest points more prominent. Furthermore, CiteSpace may be a controversial tool and it is constantly being improved. We just directly described the top subjects, sketched the broad outline of this field as intuitively as possible and obtained an overview of potential markers on PNDs, rather than a list for certain specific markers. Thus, the depth was possibly insufficient and many details of articles were not summarized adequately.

## Conclusion

In general, the issue of neurocognitive disorders after cardiac surgery has received significant attention in past 20 years. Many outstanding institutions in United States such as Duke University, Harvard Medical School and University of Pennsylvania have made great amount of excellent academic achievements in this field. Most of these publications are associated with cardiothoracic surgery and anesthesiology, but they also involve nutrition, endocrinology, molecular and cellular biology and neuroscience. From this perspective, multi-institutional cooperation and interdisciplinary collaboration are crucial to the progress and development of this scientific area.

Taken together, the most frequently cited literature comprise the basis of this field. According to the cluster analysis and timeline view, the knowledge base can be divided into four thematic domains: (1) the classification, definition and assessment of every neurocognitive disorder during cardiac surgery (e.g., PNDs, POD, and POCD), (2) the types of cardiac diseases and corresponding operation or anesthesia (e.g., congenital heart disease, CABG, and fentanyl-induced), (3) the risk factors and possible prophylactic measures (e.g., obese individual and RIPC), (4) the predictive markers and potential pathophysiology (e.g., S100b, NSE, and KYNA). Obviously, three classic biomarkers of S100b, NSE, and KYNA were frequently studied by researchers in past 20 years.

As far as the research focuses are concerned, the co-occurring keywords with high frequency can represent various scientific hotspots in the field. They include several pivotal topics: cardiac disease and surgery, short- or long-term postoperative outcomes, brain injury and cognitive dysfunction, risk factors and complex relationship among possible events. From the burst analysis of co-occurring keywords and co-cited references, we get to know that the main research hotspots of this field have transferred from hemodynamic changes and mechanisms during kinds of cardiac surgery, release of markers associated with tissue damage (2001–2010), promotion of non-technical operation skills and teamwork, improvement of operation patterns and protection of multiple organs (2008–2018) to standardized management and individualized treatment, inflammation-related mechanisms and pathways (2018–2021).

The citations of articles published in recent year are usually not very high due to their short time. Therefore, we performed the structural variation analysis. From the results of it, 15 potentially transformative articles published in 2019–2021 have three main themes: neuromonitoring such as cerebral oxygen saturation, perfusion and the depth of anesthesia in the operation, comprehensive management and prevention of delirium, and diagnosis with biomarkers of PNDs involving neurovascular or immune mechanisms. To some extent, these topics are likely to be the research frontiers. In addition, combined with the results of the burst keywords and the burst references in recent years, the research of inflammation between cardiac diseases or surgery and the research applying the nomenclature of PNDs are the currently main research directions.

The current review examined valuable diagnostic and prognostic markers for treating neurocognitive disorders after cardiac surgery via CiteSpace, which is a convenient citation visual analysis tool for researchers to quickly and objectively understand the foundation, status, hotspots and frontiers of a scientific field. In this context, we can attempt to impersonally describe what we saw and what we knew according to the results. Rather, with the purpose of answering a defined question or proving a certain assumption, some traditional systematic reviews and meta-analysis tend to subjectively filter the articles, explain the results from their own points of view, and then unavoidably introduced more potential bias. Compared with those approaches, CiteSpace, a more efficient way, can provide multi-faceted and multi-dimensional information of a research field based on massive publications, for example, the distribution of institutions/countries or journals, the valuable references and hot keywords, the evolution of main focuses and so on. We can grasp the whole developing tendency of a field on a macro level and understand the temporal and spatial characteristics of this area. However, being limited to the specific search strategy, different database and diverse parameter settings, the results may vary in some details, so the interpretation should be cautious.

## Data Availability Statement

The raw data supporting the conclusions of this article will be made available by the authors, without undue reservation.

## Author Contributions

LJ: design of the study, analysis, writing, and revision of the article. FL: design of the study and revision of the article. Both authors contributed to the article and approved the submitted version.

## Funding

The research has received funding from Excellent Talents Training Grant Program of Xicheng District, Beijing, China (202153).

## Conflict of Interest

The authors declare that the research was conducted in the absence of any commercial or financial relationships that could be construed as a potential conflict of interest.

## Publisher's Note

All claims expressed in this article are solely those of the authors and do not necessarily represent those of their affiliated organizations, or those of the publisher, the editors and the reviewers. Any product that may be evaluated in this article, or claim that may be made by its manufacturer, is not guaranteed or endorsed by the publisher.
