# Supplementary figures and images for: Potential Markers of Neurocognitive Disorders After Cardiac Surgery: A Bibliometric and Visual Analysis
Source: Front Aging Neurosci. 2022 Jun 1;14:868158. doi: 10.3389/fnagi.2022.868158 (PMC9199578; doi:10.3389/fnagi.2022.868158)

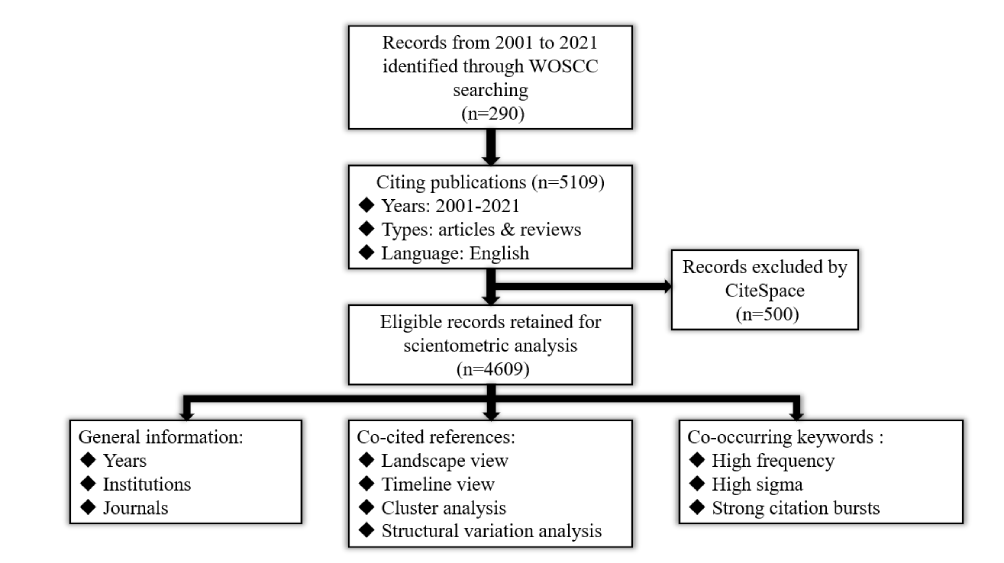

Supplement: Supplementary file 1 [file Data_Sheet_1.zip › Figure e-1The study flow diagram.tiff]

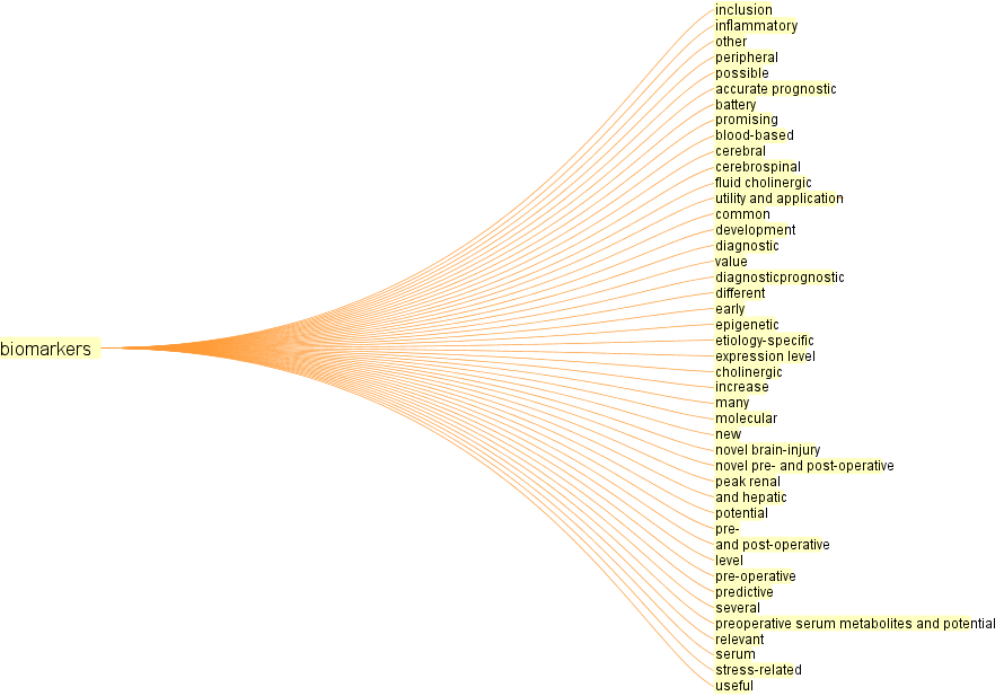

Supplement: Supplementary file 1 [file Data_Sheet_1.zip › Figure e-2(A) The key concepts selected from citing articles of Cluster #0.tiff]

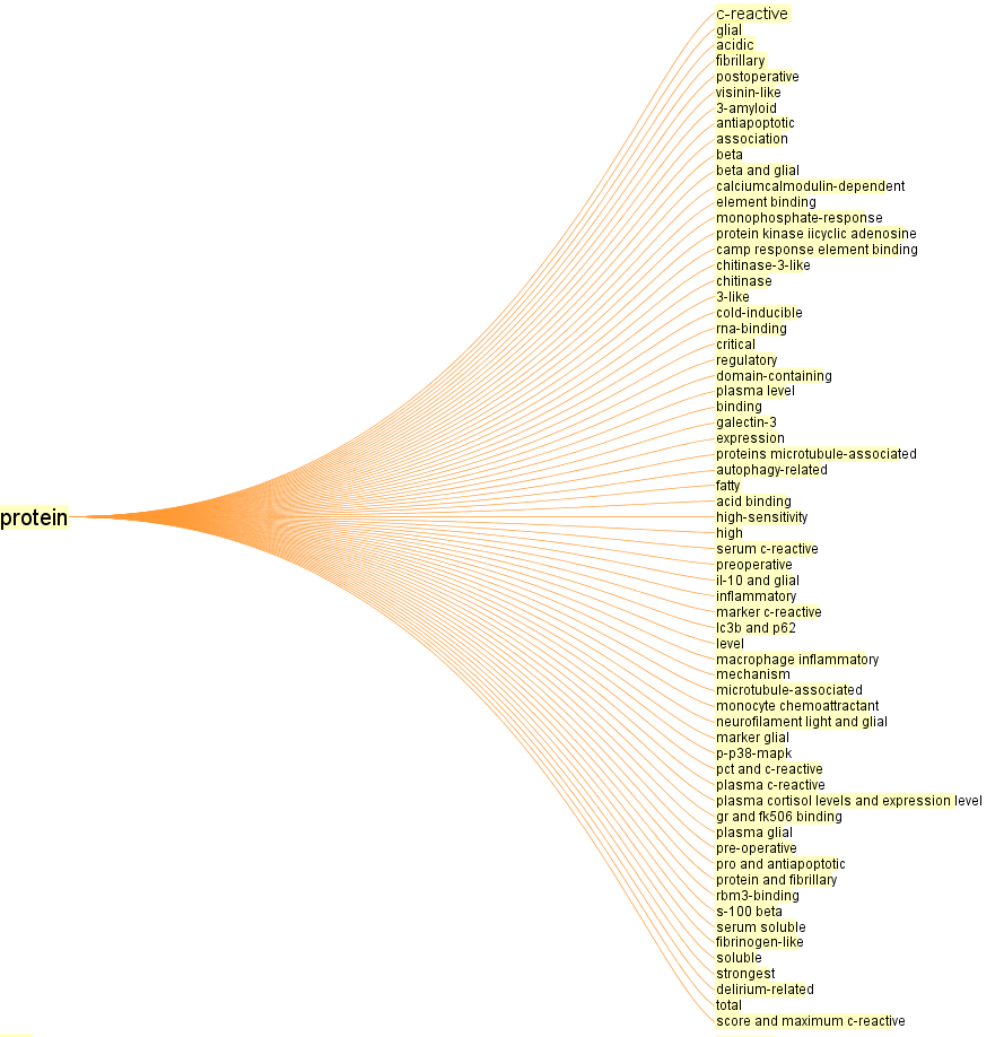

Supplement: Supplementary file 1 [file Data_Sheet_1.zip › Figure e-2(B) The key concepts selected from citing articles of Cluster #0.tiff]
